# 2-Chloro­methyl-3-methyl-1-phenyl­sulfonyl-1*H*-indole

**DOI:** 10.1107/S1600536809041191

**Published:** 2009-10-17

**Authors:** B. Saravanan, V. Dhayalan, A. K. Mohanakrishnan, G. Chakkaravarthi, V. Manivannan

**Affiliations:** a Department of Research and Development, PRIST University, Vallam, Thanjavur 613 403, Tamil Nadu, India; b Department of Organic Chemistry, University of Madras, Guindy Campus, Chennai 600 025, India; c Department of Physics, CPCL Polytechnic College, Chennai 600 068, India

## Abstract

In the title compound, C_16_H_14_ClNO_2_S, the phenyl ring makes a dihedral angle of 78.1 (1)° with the indole ring system. The mol­ecular structure is stabilized by weak intra­molecular C—H⋯O inter­actions. The crystal structure exhibits weak inter­molecular C—H⋯O, C—H⋯π and π–π [centroid–centroid distances = 3.620 (1)–3.794 (1) Å] inter­actions.

## Related literature

For the biological activity of indole derivatives, see: Okabe & Adachi (1998 ▶); Schollmeyer *et al.* (1995 ▶). For related crystal structures, see: Chakkaravarthi *et al.* (2007 ▶, 2008 ▶).
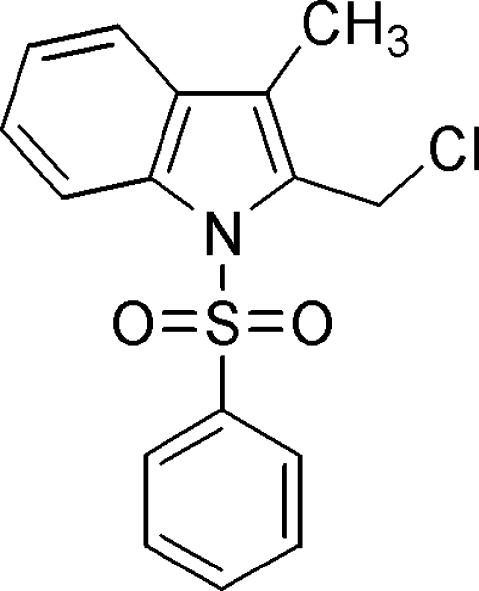


## Experimental

### 

#### Crystal data

C_16_H_14_ClNO_2_S
*M* *_r_* = 319.79
Monoclinic, 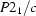

*a* = 7.9769 (6) Å
*b* = 10.8064 (9) Å
*c* = 17.3418 (12) Å
β = 97.500 (2)°
*V* = 1482.1 (2) Å^3^
*Z* = 4
Mo *K*α radiation
μ = 0.40 mm^−1^
*T* = 295 K
0.30 × 0.28 × 0.26 mm

#### Data collection

Bruker Kappa APEXII diffractometer
Absorption correction: multi-scan (**SADABS**; Sheldrick, 1996 ▶) *T*_min_ = 0.889, *T*_max_ = 0.903
17616 measured reflections
3885 independent reflections
3201 reflections with *I* > 2σ(*I*)
*R*_int_ = 0.024

#### Refinement

*R*[*F* ^2^ > 2σ(*F* ^2^)] = 0.041
*wR*(*F* ^2^) = 0.120
*S* = 1.04
3885 reflections
191 parameters
H-atom parameters constrained
Δρ_max_ = 0.59 e Å^−3^
Δρ_min_ = −0.45 e Å^−3^

### 

Data collection: *APEX2* (Bruker, 2004 ▶); cell refinement: *SAINT* (Bruker, 2004 ▶); data reduction: *SAINT*; program(s) used to solve structure: *SHELXS97* (Sheldrick, 2008 ▶); program(s) used to refine structure: *SHELXL97* (Sheldrick, 2008 ▶); molecular graphics: *PLATON* (Spek, 2009 ▶); software used to prepare material for publication: *SHELXL97*.

## Supplementary Material

Crystal structure: contains datablocks global, I. DOI: 10.1107/S1600536809041191/is2470sup1.cif

Structure factors: contains datablocks I. DOI: 10.1107/S1600536809041191/is2470Isup2.hkl

Additional supplementary materials:  crystallographic information; 3D view; checkCIF report

## Figures and Tables

**Table 1:** Hydrogen-bond geometry (Å, °)

*D*—H⋯*A*	*D*—H	H⋯*A*	*D*⋯*A*	*D*—H⋯*A*
C2—H2⋯O1	0.93	2.51	2.886 (3)	104
C13—H13⋯O1	0.93	2.47	3.033 (2)	119
C15—H15*B*⋯O2	0.97	2.31	2.853 (3)	114
C16—H16*A*⋯*Cg*2^ii^	0.96	2.94	3.777 (2)	146
C16—H16*B*⋯*Cg*3^iii^	0.96	2.92	3.781 (3)	149

Symmetry codes: (i) 
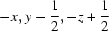
; (ii) 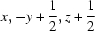; (iii) 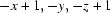. *Cg*2 and *Cg*3 are the centroids of the C1–C6 and C9–C14 rings, respectively.

**Fig. 1.**
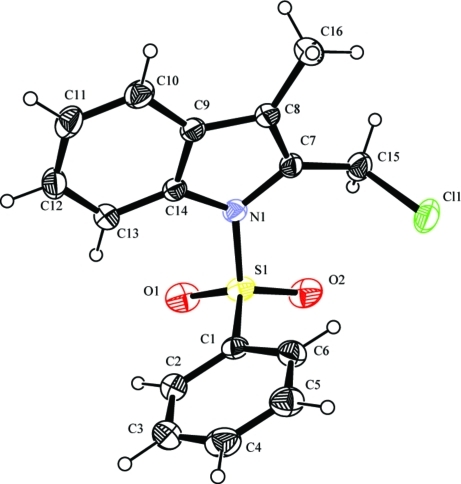
The molecular structure of (I), with atom labels and 30% probability displacement ellipsoids for non-H atoms.

**Fig. 2.**
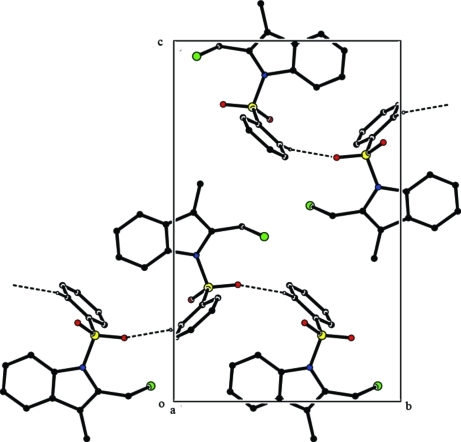
The packing of (I), viewed down the a axis. Hydrogen bonds are shown as dashed lines. H atoms not involved in hydrogen bonding have been omitted.

## supplementary crystallographic information

### Comment

The indole derivatives are found to possess antibacterial (Okabe & Adachi, 1998) and antitumour (Schollmeyer *et al.*, 1995) activities. In continuation of our studies in indole derivatives, we present the crystal structure of the title compound (I). The geometric parameters of (I) (Fig. 1) agree with those in the reported structures (Chakkaravarthi *et al.*, 2007, 2008).

The plane of the phenyl ring forms a dihedral angle of 78.1 (1)° with the indole ring system. The torsion angles O2—S1—N1—C7 and O1—S1—N1—C14 [-22.9 (2)° and 54.5 (1)°, respectively] indicate the *syn*-conformation of the sulfonyl moiety. The sum of bond angles around N1 [355.6 (1)°] indicates that N1 is *sp*^2^-hybridized.

The molecular packing is stabilized by weak intramolecular C—H···O interactions and the crystal packing of I (Fig. 2) exhibit weak intermolecular C—H···O, C—H···π (see Table 1) and π–π [*Cg*1···*Cg*1(-*x*, -*y*, 1 - *z*) distance of 3.620 (1) Å and *Cg*1···*Cg*3 (-*x*, -*y*, 1 - *z*) distance of 3.794 (1) Å interactions. *Cg*1 and *Cg*3 are the centroids of the N1/C7–C9/C14 and C9–C14 rings, respectively.

### Experimental

A mixture of 1-phenylsulfonyl-2,3-dimethylindole (5 g, 17.5 mmol) and finely powdered NCS (2.56 g, 19.17 mmol) in dry CCl_4_ (80 ml) containing catalytic amount of benzoyl peroxide (0.1 g) was refluxed for 1 h and cooled. The floated succinimide was filtered off and washed with CCl_4_ (15 ml). The solvent was then removed completely under vacuo and recrystallized from CDCl_3_.

### Refinement

H atoms were positioned geometrically and refined using riding model with C—H = 0.93 Å and *U*_iso_(H) = 1.2*U*_eq_(C) for aromatic C—H, C—H = 0.97 Å and *U*_iso_(H) = 1.5*U*_eq_(C) for methylene, and C—H = 0.96 Å and *U*_iso_(H) = 1.5*U*_eq_(C) for methyl.

### Crystal data

**Table:**

C_16_H_14_ClNO_2_S	*F*(000) = 664
*M**_r_* = 319.79	*D*_x_ = 1.433 Mg m^−^^3^
Monoclinic, *P*2_1_/*c*	Mo *K*α radiation, λ = 0.71073 Å
Hall symbol: -P 2ybc	Cell parameters from 8432 reflections
*a* = 7.9769 (6) Å	θ = 2.2–28.8°
*b* = 10.8064 (9) Å	µ = 0.40 mm^−^^1^
*c* = 17.3418 (12) Å	*T* = 295 K
β = 97.500 (2)°	Block, colourless
*V* = 1482.1 (2) Å^3^	0.30 × 0.28 × 0.26 mm
*Z* = 4	

### Data collection

**Table:**

Bruker Kappa APEXII diffractometer	3885 independent reflections
Radiation source: fine-focus sealed tube	3201 reflections with *I* > 2σ(*I*)
graphite	*R*_int_ = 0.024
ω and φ scans	θ_max_ = 28.9°, θ_min_ = 2.2°
Absorption correction: multi-scan (*SADABS*; Sheldrick, 1996)	*h* = −10→10
*T*_min_ = 0.889, *T*_max_ = 0.903	*k* = −14→14
17616 measured reflections	*l* = −13→23

### Refinement

**Table:**

Refinement on *F*^2^	Primary atom site location: structure-invariant direct methods
Least-squares matrix: full	Secondary atom site location: difference Fourier map
*R*[*F*^2^ > 2σ(*F*^2^)] = 0.041	Hydrogen site location: inferred from neighbouring sites
*wR*(*F*^2^) = 0.120	H-atom parameters constrained
*S* = 1.04	*w* = 1/[σ^2^(*F*_o_^2^) + (0.0606*P*)^2^ + 0.5623*P*] where *P* = (*F*_o_^2^ + 2*F*_c_^2^)/3
3885 reflections	(Δ/σ)_max_ < 0.001
191 parameters	Δρ_max_ = 0.59 e Å^−^^3^
0 restraints	Δρ_min_ = −0.45 e Å^−^^3^

### Fractional atomic coordinates and isotropic or equivalent isotropic displacement parameters (Å^2^)

**Table:**

	*x*	*y*	*z*	*U*_iso_*/*U*_eq_	
C1	0.2222 (2)	0.12567 (15)	0.26775 (9)	0.0376 (3)	
C2	0.2156 (3)	0.03299 (18)	0.21251 (11)	0.0510 (4)	
H2	0.1172	−0.0125	0.1989	0.061*	
C3	0.3596 (3)	0.0096 (2)	0.17795 (12)	0.0633 (6)	
H3	0.3576	−0.0519	0.1403	0.076*	
C4	0.5042 (3)	0.0757 (2)	0.19847 (13)	0.0602 (5)	
H4	0.6006	0.0573	0.1758	0.072*	
C5	0.5086 (3)	0.1686 (2)	0.25200 (12)	0.0554 (5)	
H5	0.6069	0.2145	0.2647	0.067*	
C6	0.3671 (2)	0.19466 (18)	0.28736 (10)	0.0460 (4)	
H6	0.3695	0.2578	0.3239	0.055*	
C7	0.1768 (2)	0.16719 (15)	0.47207 (10)	0.0391 (3)	
C8	0.2587 (2)	0.08731 (16)	0.52391 (9)	0.0408 (4)	
C9	0.2430 (2)	−0.03433 (15)	0.49110 (9)	0.0371 (3)	
C10	0.3019 (3)	−0.14938 (18)	0.51880 (11)	0.0498 (4)	
H10	0.3646	−0.1568	0.5677	0.060*	
C11	0.2658 (3)	−0.25159 (18)	0.47269 (13)	0.0543 (5)	
H11	0.3054	−0.3288	0.4904	0.065*	
C12	0.1713 (3)	−0.24139 (17)	0.40010 (12)	0.0503 (4)	
H12	0.1491	−0.3121	0.3700	0.060*	
C13	0.1090 (2)	−0.12916 (16)	0.37131 (10)	0.0426 (4)	
H13	0.0446	−0.1229	0.3227	0.051*	
C14	0.14676 (19)	−0.02597 (15)	0.41808 (9)	0.0343 (3)	
C15	0.1467 (3)	0.29996 (17)	0.48501 (12)	0.0530 (5)	
H15A	0.1376	0.3130	0.5396	0.064*	
H15B	0.0398	0.3236	0.4555	0.064*	
C16	0.3463 (3)	0.1166 (2)	0.60340 (11)	0.0604 (5)	
H16A	0.3348	0.2033	0.6137	0.091*	
H16B	0.4640	0.0960	0.6062	0.091*	
H16C	0.2963	0.0694	0.6414	0.091*	
N1	0.10231 (17)	0.09922 (13)	0.40560 (7)	0.0370 (3)	
O1	−0.08880 (16)	0.07654 (16)	0.28192 (8)	0.0602 (4)	
O2	0.0208 (2)	0.28227 (14)	0.32369 (9)	0.0621 (4)	
S1	0.04580 (5)	0.15288 (4)	0.31625 (2)	0.04245 (13)	
Cl1	0.31210 (9)	0.39887 (5)	0.45712 (4)	0.07026 (19)	

### Atomic displacement parameters (Å^2^)

**Table:**

	*U*^11^	*U*^22^	*U*^33^	*U*^12^	*U*^13^	*U*^23^
C1	0.0415 (8)	0.0392 (8)	0.0318 (7)	0.0045 (6)	0.0032 (6)	0.0087 (6)
C2	0.0614 (11)	0.0464 (10)	0.0462 (9)	−0.0100 (9)	0.0108 (8)	−0.0018 (8)
C3	0.0900 (16)	0.0496 (12)	0.0557 (12)	−0.0003 (11)	0.0294 (11)	−0.0080 (9)
C4	0.0614 (12)	0.0649 (13)	0.0592 (12)	0.0082 (10)	0.0270 (10)	0.0068 (10)
C5	0.0454 (9)	0.0698 (14)	0.0519 (10)	−0.0043 (9)	0.0090 (8)	0.0039 (9)
C6	0.0467 (9)	0.0519 (10)	0.0389 (8)	−0.0010 (8)	0.0040 (7)	−0.0004 (7)
C7	0.0465 (9)	0.0336 (8)	0.0401 (8)	−0.0044 (7)	0.0162 (7)	−0.0023 (6)
C8	0.0469 (9)	0.0415 (9)	0.0347 (7)	−0.0092 (7)	0.0082 (6)	−0.0005 (6)
C9	0.0391 (8)	0.0374 (8)	0.0349 (7)	−0.0027 (6)	0.0058 (6)	0.0039 (6)
C10	0.0539 (10)	0.0460 (10)	0.0470 (9)	0.0006 (8)	−0.0025 (8)	0.0117 (8)
C11	0.0630 (12)	0.0355 (9)	0.0649 (12)	0.0069 (8)	0.0097 (9)	0.0094 (8)
C12	0.0585 (11)	0.0359 (9)	0.0579 (11)	−0.0027 (8)	0.0130 (9)	−0.0072 (8)
C13	0.0474 (9)	0.0413 (9)	0.0392 (8)	−0.0026 (7)	0.0057 (7)	−0.0033 (7)
C14	0.0355 (7)	0.0338 (8)	0.0344 (7)	0.0006 (6)	0.0081 (6)	0.0029 (6)
C15	0.0683 (12)	0.0373 (9)	0.0588 (11)	−0.0017 (9)	0.0279 (9)	−0.0040 (8)
C16	0.0773 (14)	0.0624 (13)	0.0397 (9)	−0.0185 (11)	0.0007 (9)	−0.0052 (9)
N1	0.0423 (7)	0.0337 (7)	0.0354 (6)	0.0033 (5)	0.0070 (5)	0.0039 (5)
O1	0.0372 (6)	0.0834 (11)	0.0567 (8)	0.0000 (7)	−0.0064 (6)	0.0106 (7)
O2	0.0714 (10)	0.0515 (9)	0.0644 (9)	0.0286 (7)	0.0124 (7)	0.0183 (7)
S1	0.0366 (2)	0.0485 (3)	0.0416 (2)	0.01086 (17)	0.00270 (15)	0.01142 (17)
Cl1	0.0967 (4)	0.0386 (3)	0.0815 (4)	−0.0169 (3)	0.0344 (3)	−0.0031 (2)

### Geometric parameters (Å, °)

**Table:**

C1—C6	1.380 (3)	C10—C11	1.372 (3)
C1—C2	1.382 (3)	C10—H10	0.9300
C1—S1	1.7559 (17)	C11—C12	1.385 (3)
C2—C3	1.387 (3)	C11—H11	0.9300
C2—H2	0.9300	C12—C13	1.379 (3)
C3—C4	1.364 (3)	C12—H12	0.9300
C3—H3	0.9300	C13—C14	1.389 (2)
C4—C5	1.365 (3)	C13—H13	0.9300
C4—H4	0.9300	C14—N1	1.408 (2)
C5—C6	1.382 (3)	C15—Cl1	1.812 (2)
C5—H5	0.9300	C15—H15A	0.9700
C6—H6	0.9300	C15—H15B	0.9700
C7—C8	1.352 (2)	C16—H16A	0.9600
C7—N1	1.429 (2)	C16—H16B	0.9600
C7—C15	1.477 (2)	C16—H16C	0.9600
C8—C9	1.431 (2)	N1—S1	1.6607 (13)
C8—C16	1.496 (2)	O1—S1	1.4216 (15)
C9—C10	1.392 (2)	O2—S1	1.4206 (15)
C9—C14	1.395 (2)		
			
C6—C1—C2	121.15 (17)	C12—C11—H11	119.5
C6—C1—S1	119.29 (13)	C13—C12—C11	121.79 (18)
C2—C1—S1	119.53 (14)	C13—C12—H12	119.1
C1—C2—C3	118.21 (19)	C11—C12—H12	119.1
C1—C2—H2	120.9	C12—C13—C14	117.02 (17)
C3—C2—H2	120.9	C12—C13—H13	121.5
C4—C3—C2	120.80 (19)	C14—C13—H13	121.5
C4—C3—H3	119.6	C13—C14—C9	121.98 (15)
C2—C3—H3	119.6	C13—C14—N1	130.59 (15)
C3—C4—C5	120.56 (19)	C9—C14—N1	107.42 (13)
C3—C4—H4	119.7	C7—C15—Cl1	113.22 (13)
C5—C4—H4	119.7	C7—C15—H15A	108.9
C4—C5—C6	120.1 (2)	Cl1—C15—H15A	108.9
C4—C5—H5	119.9	C7—C15—H15B	108.9
C6—C5—H5	119.9	Cl1—C15—H15B	108.9
C1—C6—C5	119.14 (18)	H15A—C15—H15B	107.7
C1—C6—H6	120.4	C8—C16—H16A	109.5
C5—C6—H6	120.4	C8—C16—H16B	109.5
C8—C7—N1	108.80 (14)	H16A—C16—H16B	109.5
C8—C7—C15	126.40 (17)	C8—C16—H16C	109.5
N1—C7—C15	124.29 (16)	H16A—C16—H16C	109.5
C7—C8—C9	108.21 (15)	H16B—C16—H16C	109.5
C7—C8—C16	127.22 (17)	C14—N1—C7	107.46 (13)
C9—C8—C16	124.53 (17)	C14—N1—S1	120.74 (11)
C10—C9—C14	119.47 (16)	C7—N1—S1	127.36 (12)
C10—C9—C8	132.44 (16)	O2—S1—O1	120.13 (10)
C14—C9—C8	108.08 (14)	O2—S1—N1	106.45 (8)
C11—C10—C9	118.82 (17)	O1—S1—N1	106.64 (8)
C11—C10—H10	120.6	O2—S1—C1	109.81 (9)
C9—C10—H10	120.6	O1—S1—C1	108.11 (9)
C10—C11—C12	120.91 (18)	N1—S1—C1	104.57 (7)
C10—C11—H11	119.5		
			
C6—C1—C2—C3	1.0 (3)	C10—C9—C14—N1	179.02 (15)
S1—C1—C2—C3	−176.87 (15)	C8—C9—C14—N1	−0.22 (18)
C1—C2—C3—C4	0.5 (3)	C8—C7—C15—Cl1	91.1 (2)
C2—C3—C4—C5	−1.8 (4)	N1—C7—C15—Cl1	−98.03 (19)
C3—C4—C5—C6	1.6 (3)	C13—C14—N1—C7	−179.07 (16)
C2—C1—C6—C5	−1.2 (3)	C9—C14—N1—C7	1.18 (17)
S1—C1—C6—C5	176.64 (15)	C13—C14—N1—S1	−21.1 (2)
C4—C5—C6—C1	−0.1 (3)	C9—C14—N1—S1	159.16 (11)
N1—C7—C8—C9	1.60 (19)	C8—C7—N1—C14	−1.75 (17)
C15—C7—C8—C9	173.60 (16)	C15—C7—N1—C14	−173.96 (15)
N1—C7—C8—C16	−176.32 (18)	C8—C7—N1—S1	−157.84 (13)
C15—C7—C8—C16	−4.3 (3)	C15—C7—N1—S1	30.0 (2)
C7—C8—C9—C10	−179.98 (19)	C14—N1—S1—O2	−176.12 (13)
C16—C8—C9—C10	−2.0 (3)	C7—N1—S1—O2	−22.86 (16)
C7—C8—C9—C14	−0.87 (19)	C14—N1—S1—O1	54.50 (14)
C16—C8—C9—C14	177.12 (17)	C7—N1—S1—O1	−152.24 (14)
C14—C9—C10—C11	1.1 (3)	C14—N1—S1—C1	−59.89 (14)
C8—C9—C10—C11	−179.89 (19)	C7—N1—S1—C1	93.37 (15)
C9—C10—C11—C12	−0.6 (3)	C6—C1—S1—O2	43.32 (16)
C10—C11—C12—C13	−0.3 (3)	C2—C1—S1—O2	−138.81 (15)
C11—C12—C13—C14	0.6 (3)	C6—C1—S1—O1	176.10 (14)
C12—C13—C14—C9	−0.1 (2)	C2—C1—S1—O1	−6.02 (17)
C12—C13—C14—N1	−179.82 (17)	C6—C1—S1—N1	−70.55 (15)
C10—C9—C14—C13	−0.8 (2)	C2—C1—S1—N1	107.33 (15)
C8—C9—C14—C13	180.00 (15)		

### Hydrogen-bond geometry (Å, °)

**Table:**

*D*—H···*A*	*D*—H	H···*A*	*D*···*A*	*D*—H···*A*
C2—H2···O1	0.93	2.51	2.886 (3)	104
C13—H13···O1	0.93	2.47	3.033 (2)	119
C15—H15B···O2	0.97	2.31	2.853 (3)	114
C2—H2···O2^i^	0.93	2.48	3.314 (2)	149
C16—H16A···Cg2^ii^	0.96	2.94	3.777 (2)	146
C16—H16B···Cg3^iii^	0.96	2.92	3.781 (3)	149

Symmetry codes: (i) −*x*, *y*−1/2, −*z*+1/2; (ii) *x*, −*y*+1/2, *z*+1/2; (iii) −*x*+1, −*y*, −*z*+1.
